# Supplementary material for: Interaction and influence of a flow field and particleboard particles in an airflow forming machine with a coupled Euler-DPM model
Source: PLoS One. 2021 Jun 17;16(6):e0253311. doi: 10.1371/journal.pone.0253311 (PMC8211206; doi:10.1371/journal.pone.0253311)
Supplement: S1 File — (PDF) [file pone.0253311.s001.pdf]

Tabular data

Table 1 Comparison of simulated and experimental values

Measuring point number	Test value/($m•s^{-1}$)	Numerical value/($m•s^{-1}$)	Error/%
1	2.05	2.10	2.44
2	2.38	2.41	1.26
3	2.56	2.59	1.17
4	2.47	2.48	0.40
5	2.64	2.64	0
6	2.60	2.55	1.92
7	2.20	2.19	0.45

Table 2 Different negative pressure fan parameters and outlet speeds

Model	Fan frequency/*Hz*	Fan air volume/$m^3•h^{-1}$	Outlet speed/$m•s^{-1}$
Original model	27	12065.8	24.7
Increasing air volume	45.7	19543.7	40
Decreasing air volume	15.2	7328.8	15

Table 3 Geometric parameters of different models

Model	L_1/(mm)	h_1/(mm)	h_2/(mm)	L/(mm)
Model 1	1870	310	230	5300
Model 2	1370	310	230	5300
Model 3	2370	310	230	5300
Model 4	2570	310	230	5300
Model 5	1870	310	0	5300

Figure data

Fig 10. Wind speed comparison of different models

X(Measuring points)	Y1(Original model)	Y2(Decrease air volume model)	Y3(Increase air volume model)
1	2.10026	1.34288	2.75042
2	2.40462	1.36162	2.8997
3	2.58825	1.41598	2.87438
4	2.47659	1.46666	2.82455
5	2.64154	1.8	2.79549
6	2.54717	1.4	2.77503
7	2.18699	1.3	2.786
1	2.10026	1.34288	2.75042

Fig 19. Particle diameter distribution

Fig 19(a)

X(Particle diameter)	Y(Percentage)
0.0002	0.02152
0.00029	0.24193
0.00038	1.47272
0.00047	6.02417
0.00056	17.5142
0.00064	33.24084
0.00073	31.68699
0.00082	9.39752
0.00091	0.39953
0.001	0.00058

Fig 19(b)

X(Particle diameter)	Y(Percentage)
0.0002	2.01426
0.00029	4.80328

0.00038	8.68448
0.00047	12.91654
0.00056	16.24744
0.00064	17.36335
0.00073	15.64597
0.00082	11.71289
0.00091	7.14326
0.001	3.46853

Fig 22. Comparison of the characteristic line speed of the same section

Fig 22(a)

X(Coordinate)	Y1(Line-1)	Y2(Line-1*)
-2.64041	2.7461	0.9
-2.5496	3.13868	0.75636
-2.45879	3.16845	0.57656
-2.36798	3.22893	0.63911
-2.27717	3.28011	0.68375
-2.18636	3.1887	0.63841
-2.09555	3.07321	0.67831
-2.00474	3.13609	0.63249
-1.91393	2.97371	0.69515
-1.82312	2.76384	0.87166
-1.73231	2.71194	0.76167
-1.6415	2.54705	0.73621
-1.55069	2.52551	0.83256
-1.45988	2.46156	0.76086
-1.36907	2.44465	0.66301
-1.27826	2.37029	0.60321
-1.18745	2.38942	0.66873

-1.09664	2.44581	0.86759
-1.00583	2.33071	0.84689
-0.91502	2.31527	0.60812
-0.82421	2.36711	0.56626
-0.7334	2.46537	0.6137
-0.64259	2.49959	0.70123
-0.55179	2.53118	0.56981
-0.46098	2.62933	0.64119
-0.37017	2.6808	0.75784
-0.27936	2.6104	0.91903
-0.18855	2.6688	0.76532
-0.09774	2.63004	0.80967
-0.00693	2.00754	1.31471

Fig 22(b)

X(Coordinate)	Y1(Line-2)	Y2(Line-2*)
-2.63902	1.65114	1.51619
-2.5484	1.34744	1.43499
-2.45779	1.15512	1.4458
-2.36717	1.09818	1.4531
-2.27656	1.09179	1.43961
-2.18594	1.05305	1.4248
-2.09533	0.99037	1.441
-2.00471	0.94338	1.4069
-1.9141	0.90667	1.43831
-1.82348	0.90472	1.40518
-1.73287	0.95055	1.35755
-1.64225	1.19719	1.3794
-1.55164	1.54812	1.4265
-1.46102	2.17628	1.47585

-1.37041	2.6206	1.48725
-1.27979	2.63705	1.44133
-1.18918	2.35556	1.38826
-1.09856	1.81194	1.40094
-1.00795	1.2551	1.37044
-0.91733	0.96425	1.3717
-0.82672	0.98374	1.30465
-0.7361	1.37711	1.24426
-0.64549	2.21018	1.28729
-0.55487	2.22004	1.34891
-0.46426	1.9303	1.38708
-0.37364	1.80606	1.4264
-0.28303	1.64395	1.45986
-0.19241	1.38836	1.50617
-0.1018	1.42336	1.57063
-0.01118	2.14153	1.81616

Fig 22(c)

X(Coordinate)	Y1(Line-3)	Y2(Line-3*)
-2.525	2.12991	0.30648
-2.45131	2.18787	0.23783
-2.37762	2.33531	0.25768
-2.30393	2.4181	0.22308
-2.23024	2.477	0.2282
-2.15655	2.48947	0.24486
-2.08286	2.48355	0.2985
-2.00917	2.50262	0.39544
-1.93548	2.56281	0.55582
-1.86179	2.47397	0.76301
-1.7881	2.66172	0.99033

-1.71441	2.604	1.23489
-1.64072	2.53148	1.46496
-1.56703	2.38721	1.65843
-1.49334	2.3548	1.84881
-1.41966	2.42817	1.98168
-1.34597	2.56388	2.12473
-1.27228	2.50214	2.22117
-1.19859	2.67216	2.32407
-1.1249	2.70716	2.38776
-1.05121	2.63288	2.40412
-0.97752	2.42517	2.36818
-0.90383	2.33434	2.27326
-0.83014	2.43079	2.17388
-0.75645	2.45968	2.00585
-0.68276	2.61773	1.83298
-0.60907	2.48708	1.56317
-0.53538	2.40806	1.25083
-0.46169	2.35079	1.09408
-0.388	2.32328	1.15944
